# Supplementary figures and images for: Mass Production of Rg1-Loaded Small Extracellular Vesicles Using a 3D Bioreactor System for Enhanced Cardioprotective Efficacy of Doxorubicin-Induced Cardiotoxicity
Source: Pharmaceutics. 2024 Apr 26;16(5):593. doi: 10.3390/pharmaceutics16050593 (PMC11126075; doi:10.3390/pharmaceutics16050593)

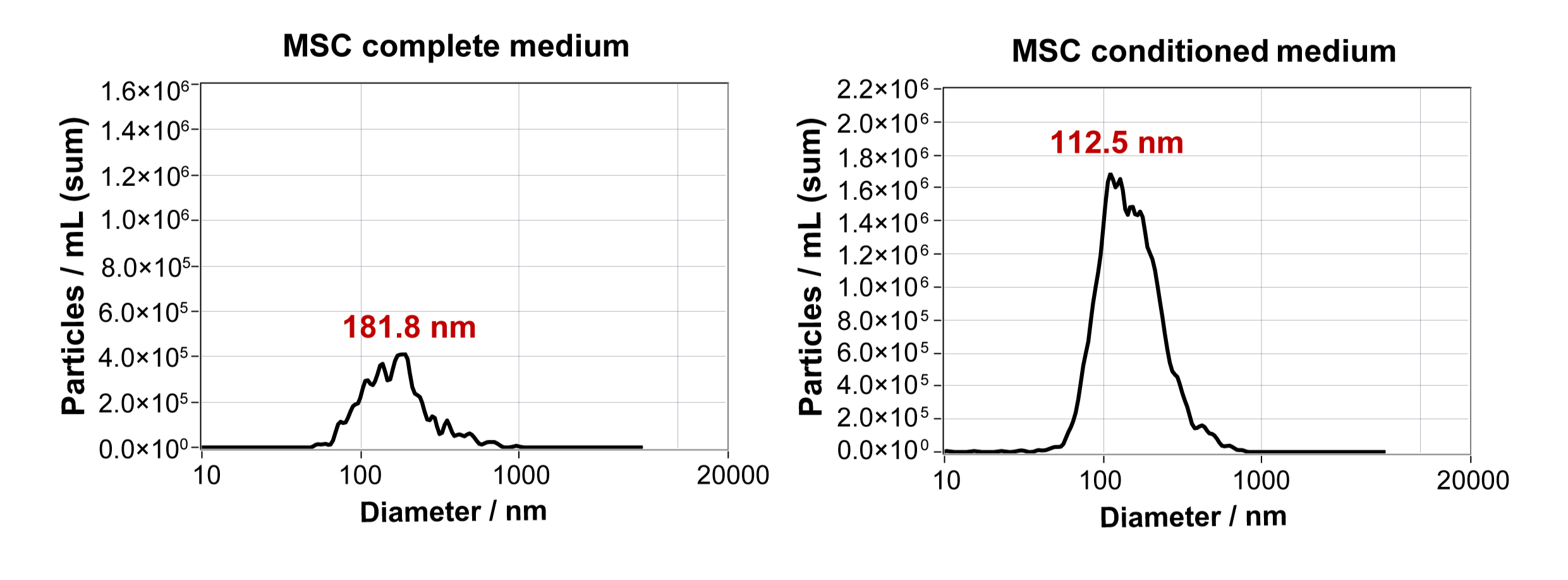

Supplement: Supplementary file 1 [file pharmaceutics-16-00593-s001.zip › Figure S1-01.tif]

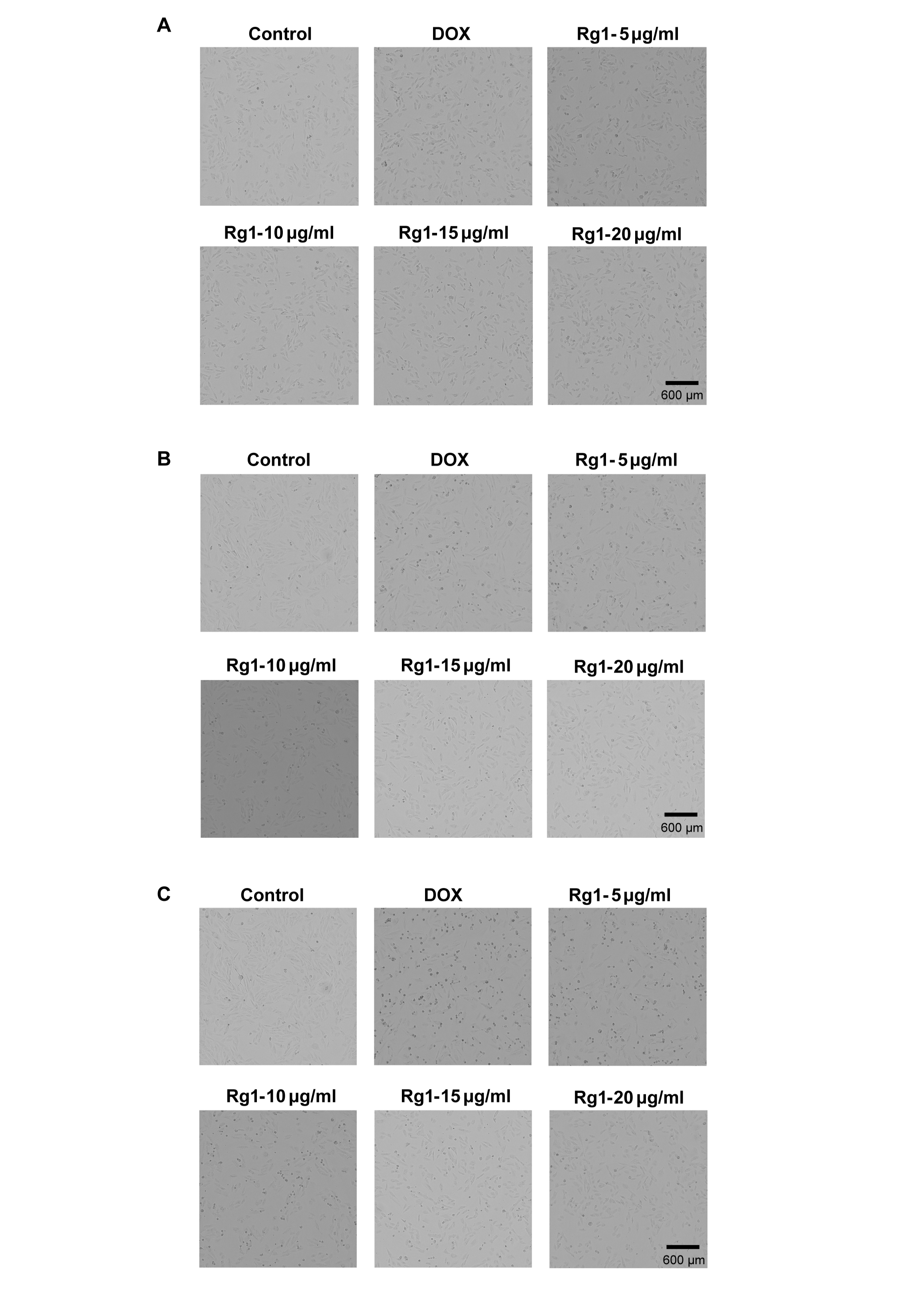

Supplement: Supplementary file 1 [file pharmaceutics-16-00593-s001.zip › Figure S2-01.tif]

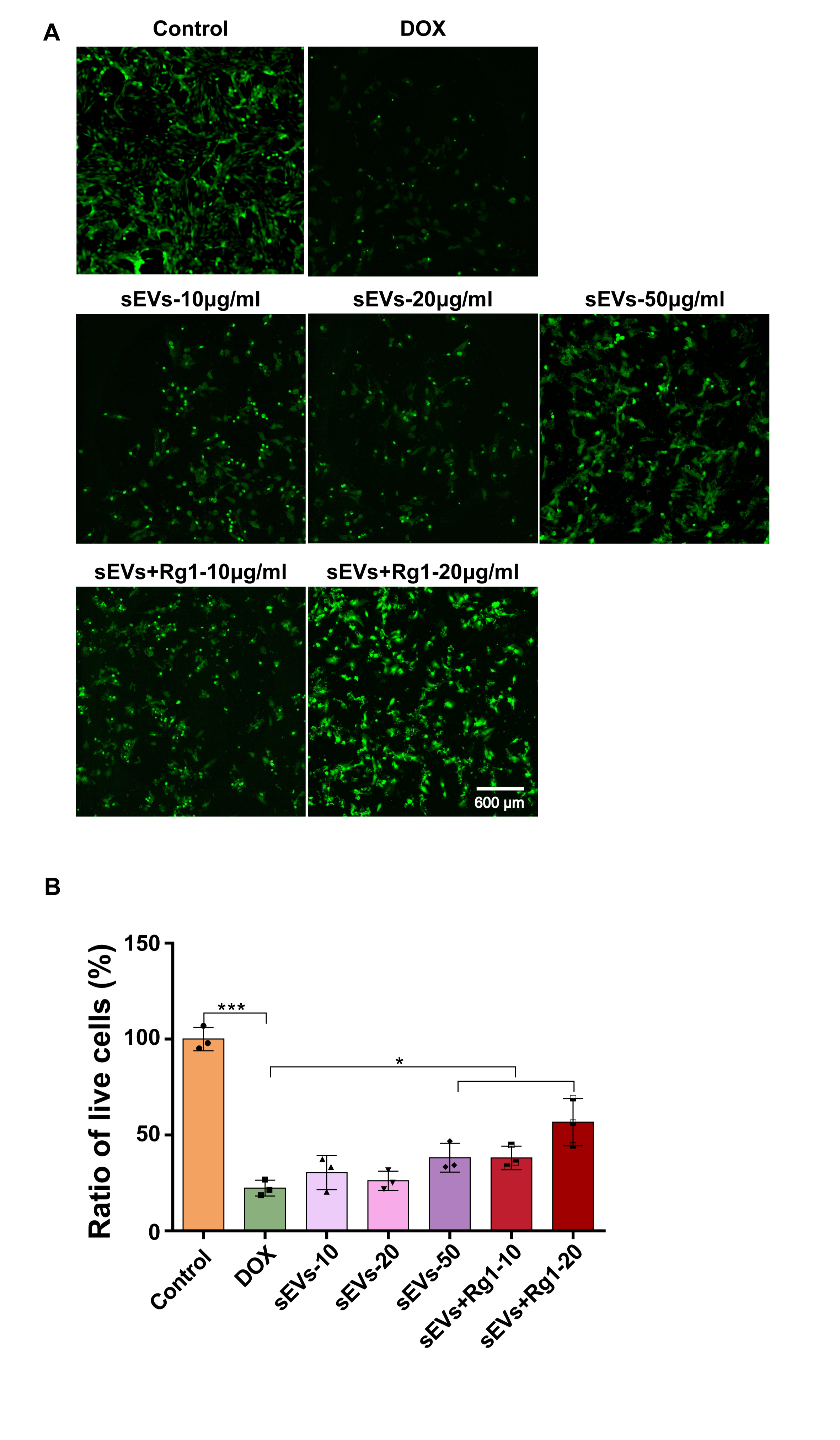

Supplement: Supplementary file 1 [file pharmaceutics-16-00593-s001.zip › Figure S3-01.tif]
